# Screening and Enzymatic Evaluation of *Saccharomyces cerevisiae* Populations from Spontaneous Fermentation of Organic Verdejo Wines

**DOI:** 10.3390/foods11213448

**Published:** 2022-10-30

**Authors:** Lorena López-Enríquez, Josefina Vila-Crespo, José Manuel Rodríguez-Nogales, Encarnación Fernández-Fernández, Violeta Ruipérez

**Affiliations:** 1 Área de Microbiología, Universidad de Valladolid, Escuela Técnica Superior de Ingenierías Agrarias, Av. Madrid 50, 34004 Palencia, Spain; 2 Área de Tecnología de los Alimentos, Universidad de Valladolid, Escuela Técnica Superior de Ingenierías Agrarias, Av. Madrid 50, 34004 Palencia, Spain

**Keywords:** enzymatic activity, *Saccharomyces cerevisiae*, strain biotyping, Verdejo wine, wine quality, yeast diversity

## Abstract

Microbial populations in spontaneous winemaking contribute to the distinctiveness and quality of the wines. In this study, molecular methods were applied to 484 isolated yeasts to survey the diversity of the *Saccharomyces cerevisiae* population in spontaneous fermentations of organic Verdejo grapes. Identification was carried out at strain level for samples from different vineyards correct.and stages of the winemaking process over the course of two vintages, establishing 54 different strains. The number of isolates belonging to each strain was not homogeneous, as two predominant strains represented more than half of the isolates independent of vineyard or vintage. Regarding the richness and abundance, differences among the stages of fermentation were confirmed, finding the highest diversity values in racked must and in the end of fermentation stages. Dissimilarity in *S. cerevisiae* communities was found among vineyards and vintages, distinguishing representative groups of isolates for each of the populations analysed. These results highlight the effect of vineyard and vintage on yeast communities as well as the presence of singular strains in populations of yeasts. Oenologically relevant enzymatic activities, β-lyase, protease and β-glucanase, were detected in 83.9%, 96.8% and 38.7% of the isolates, respectively, which may be of interest for potential future studies.

## 1. Introduction

Wine production is a complex process that involves a close association of microorganisms. The increasing interest of wine industry in the distinctiveness of their wines makes it necessary for them to understand microbial diversity and its influence on the winemaking process. Different patterns in the microbiota of grapes and must, including fungal and bacteria communities coming from the vineyard and winery, are conditioned by factors ranging from environmental conditions to winemaking practices, and they are associated with the quality, composition and aroma profile of the wine [1,2,3]. Climate and soil impact on viticulture, determining the style of wine and its quality but also affecting microbiota patterns, as factors such as wind, precipitation or temperature, among others, exert an important influence on microbial presence. In addition, viticultural and oenological practices in the wine industry also affect the presence and activity of the microorganisms involved in winemaking [4], for instance, organic grape cultivation and spontaneous fermentation constitute an important source of microbial resources and promote a wide variety of indigenous microbiota [5,6]. Recent studies have focused on highlighting the relevance of multispecies fermentations for enhancing the typicity of local white wines [7] and the capacity of those species/strains with oenologically unique properties to influence their aroma profile [8]. 

Geographical patterns of *S. cerevisiae* yeasts have been previously confirmed, suggesting that microbiota are key contributors to the regional characteristics of wines [4]. The isolation of yeasts from Pinot Noir and Chardonnay grapes from diverse vineyards showed the geographical strain diversity of *S. cerevisiae* and correlated with aroma profiles, suggesting that *S. cerevisiae* play a primary role in determining the final aromatic profile of wines [1]. Furthermore, genetic analysis of the population of *S. cerevisiae* from vineyards of six islands in the Azores Archipelago revealed the role of geography in their distribution, as divergence among vineyards of different islands was found [9]. Therefore, the influence of *S. cerevisiae* patterns on the final characteristics and the organoleptic complexity that define a singular wine evidence the requirement of ecological studies during the winemaking process. 

Alcoholic fermentation can be a spontaneous process, started and developed by indigenous yeasts, or a process driven by commercial or autochthonous starter cultures [6]. The survey of variations in grape microbiota associated with a viticultural area and the identification of indigenous yeasts for the development of potential starter cultures have emerged as strategies which confer the specific, regional character of wines [10]. The screening of indigenous *S. cerevisiae* populations enables the assessment of the effect of oenological practices on the succession and diversity of yeast populations throughout alcoholic fermentation. It also allows the identification of strains that exhibit an optimal adaptation to the environment as well as an improvement of oenological properties [11], triggering distinctive organoleptic characteristics in the wine. In this context, the enzymatic potential of yeasts is a useful tool for enhancing the organoleptic profile of wine. The limited enzymatic activity to hydrolyse aromatic precursors that *S. cerevisiae* possesses makes it a challenge to find strains implicated in the release of aromatic compounds. However, recent research has suggested that differences in volatile composition can be achieved, requiring the screening of enzymatic activity in a large number of strains. Selective culture media have been used to detect either positive or negative enzymatic activities, such as those implicated in the release of volatile organic compounds, involved in off-flavours, and those related to colour and fining and the release of toxic compounds, among other things [12,13,14,15].

Keeping up with wines of quality, health considerations are becoming a demand of consumers. A high concentration of biogenic amines (BA) has been demonstrated to cause undesirable effects related to human health [16,17]. The presence of BA in wines can be attributed to the raw materials and winemaking process, which includes BA-producing microorganisms. During alcoholic fermentation, yeasts can contribute to increasing the content of BA as a consequence of their normal metabolic activity [18]. The screening of different strains of yeasts revealed the ability of *S. cerevisiae* to produce BA, which is not a constant characteristic of the species, as strain-dependent production was found [14,16]. 

The Verdejo grape is the main variety from the appellation of origin (AO) Rueda (North Central Spain) used for the production of white wine, giving its genuineness and characteristic quality to one of the most important Spanish white wines all over the world [19]. This highlights the importance of preserving the regional distinctiveness that defines a unique and high-quality wine and is an important driving force of the Rueda region’s economy. Organic wine production with spontaneous fermentation is an appropriate combination to promote the diversity of yeast communities and, therefore, ensure regional distinctiveness. However, to our knowledge, microbial ecological studies during spontaneous fermentation in organic Verdejo grapes from the AO Rueda have not been previously reported. In this sense, exploring the microbial diversity and oenological characteristics of the indigenous yeasts in this unique white wine is of great of interest to the wine industry. 

Given the lack of studies, we surveyed the diversity of *S. cerevisiae* populations during the spontaneous fermentation of organic Verdejo grapes. Molecular methods applied to isolated yeasts from different geographical localisations and at different stages of the winemaking process were used for the identification of *S. cerevisiae* strains over the course of two vintages. The distribution and fluctuation of the yeast communities were analysed, as well as the enzymatic activities of the identified strains. Taken together, the diversity of *S. cerevisiae* strains found in conjunction with their variability in enzymatic activities highlights the importance of preserving microbial diversity for keeping the regional distinctiveness of Verdejo wine. 

## 2. Materials and Methods

### 2.1. Sampling and Yeast Isolation

Verdejo grapes were harvested from three different vineyards located in the AO Rueda during two vintages. Meteorological data are detailed in Figure S1. The work in the vineyards, belonging to Belondrade’s winery, located in the town of La Seca (Valladolid, Spain), was carried out according to ecological viticulture. Yeasts were isolated from spontaneously fermented musts in the Belondrade winery at different stages of the fermentation process (freshly crushed grape must, CM; racked must, RM; start of fermentation, SF; tumultuous fermentation, TF; end of fermentation, EF) on YPD agar containing 1% (*w/v*) yeast extract (Biolife, Milano, Italy), 2% (*w/v*) peptone (Panreac, Barcelona, Spain), 2% (*w/v*) dextrose (Scharlab, Barcelona, Spain), 2% (*w/v*) agar (Scharlab). 

### 2.2. Molecular Analysis of the Isolates

Yeasts were cultured in YPD broth at 25 °C under shaking (220 rpm) for 18–36 h, and total DNA isolation was performed [20].

Restriction fragment length polymorphism (RFLP) of the 5.8S–ITS region of ribosomal DNA (rDNA) was used for the classification and identification of the different groups of yeast isolates at species level. Amplification of the rDNA region was carried out using specific primers ITS1 (5′-TCC GTA GGT GAA CCT GCG G-3′) and ITS4 (5′-TCC TCC GCC GCT TAT TGA TAT GC-3′) (Sigma-Aldrich, Madrid, Spain) [21]. Restriction fragments were obtained by using restriction enzymes *Hae*III, *Hinf*I and *Cfo*I (10 U/µL; Fisher Scientific, Madrid, Spain). Briefly, 5 µL of the amplification product obtained was digested following the manufacturer’s recommendations. Both PCR products and their restriction fragments were resolved in 4.5% (*w/v*) D1 Low EEO agarose gels (Pronadisa, Madrid, Spain) in TAE 1X (Fisher Scientific), a current of 120 V was applied for 3 h, running a distance of approximately 5 cm. In all electrophoresis processes, the molecular weight marker GeneRuler 100 bp DNA Ladder (Fisher Scientific) was used. Post-electrophoresis staining was carried out with GelRed (Biotium Inc., Fremont, CA, USA), and gel images were acquired with the Gel Doc XR+ gel documentation system (BioRad, Hercules, CA, USA). 

In order to confirm the identifications obtained by PCR-RFLP in the 5.8S-ITS region, representative isolates from each group were analysed by sequencing of the D1/D2 region of the 28S RNA gene.

All yeast isolates identified as *S. cerevisiae* were analysed at strain level by mitochondrial DNA (mtDNA) restriction patterns [22]. Briefly, 40 µg of total DNA was digested with *Hinf*I (10 U/µL; Fisher Scientific). The digestion products were separated in 0.8% (*w/v*) agarose gels (Pronadisa). Electrophoresis was performed at 80 V for 4 h and 7.5 cm of run length, and GeneRuler 1 Kb DNA Ladder (Fisher Scientific) was used as molecular weight marker. Post-electrophoresis staining and image capture were carried out as described in the analysis of the PCR-RFLP 5.8S-ITS region. Determination and comparison of the different mtDNA restriction patterns were developed with GelComparII v6.6 software (Applied Maths, Sint-Martens-Latem, Belgium) using the Dice association coefficient and the UPGMA (unweighted pair group method using arithmetic averages) algorithm.

### 2.3. Enzymatic Activities 

#### 2.3.1. β-Lyase Activity

Β-lyase activity was determined as previously described by Belda et al. [23] in a culture medium containing 1.2% (*w/v*) yeast carbon base (Difco, Detroit, MI, USA), 0.1% (*w/v*) S-methyl-L-cysteine (Panreac), 0.01% (*w/v*) pyridoxal-5′-phosphate (Panreac) and 2% agar solution (Scharlab). A single colony was spread onto the plate’s surface and incubated at 25 °C for 72 h. Enzymatic activity was considered positive when the growth of the isolates was significant after 72 h of incubation. 

#### 2.3.2. β-Glucosidase Activity 

A culture medium containing 0.5% (*w/v*) arbutine (Sigma-Aldrich), 0.67% (*w/v*) yeast nitrogen base (Biolife), 2% (*w/v*) agar (Scharlab) and 2.0 mL of 1% (*w/v*) iron chloride (Panreac) solution for each 100 mL of medium was prepared [24]. A single colony was spread onto the plate surface and was incubated at 25 °C from 48–72 h up to 1 week if the isolates did not show growth. Dark-black cultures were considered positive. 

#### 2.3.3. β-Glucanase Activity 

The culture medium consisted of 1% (*w/v*) yeast extract (Biolife), 2% (*w/v*) peptone (Panreac), 2% (*w/v*) dextrose (Scharlab), 0.2% (*w/v*) yeast β-glucan (Megazyme) and 2% (*w/v*) agar (Scharlab) [13]. A single colony was spread onto the plates and was incubated at 25 °C for 5–7 days. Afterwards, the colonies were rinsed with distilled water, and the surface of the plate was covered with 0.03% (*w/v*) Congo red solution (Sigma-Aldrich). Isolates with positive activity showed a clear halo on the surface, which the colonies covered with their growth.

#### 2.3.4. Protease Activity 

Petri dishes were prepared with culture medium containing 1% (*w/v*) yeast extract (Biolife), 2% (*w/v*) peptone (Panreac), 2% (*w/v*) dextrose (Scharlab), 2% (*w/v*) skim milk powder (Sigma-Aldrich) and 2% (*w/v*) agar (Scharlab) [23]. A single colony was spread onto the plates and was incubated at 25 °C for 5–7 days. A clear halo around the colonies reported positive protease activity. 

#### 2.3.5. Sulfite Reductase Activity 

Broth was prepared containing 4% (*w/v*) glucose (Scharlab), 0.5% (*p/v*) ammonium sulphate (Panreac) and 1.17% (*w/v*) yeast carbon base (Difco) [23]. In 24-well culture plates, 0.5 mL of the medium was added per well, and a single colony was inoculated. A fragment of paper strips with 0.1 M lead acetate (Whatman™, Maidstone, UK) was placed on the cover of each well. The plates were incubated at 25 °C for 3 days after shaking. Paper strips with brown-black colour revealed the presence of volatile H_2_S in the upper space of the well and, therefore, positive sulfite activity. 

#### 2.3.6. Hydroxycinnamic Acid Decarboxylase (HCDC) Activity 

YPD agar supplemented with 0.145% (*p/v*) p-coumaric acid (Sigma-Aldrich) and 0.01% (*w/v*) bromocresol purple (Sigma-Aldrich) was used to evaluate HCDC activity [13]. A single colony was spread onto the plates and was incubated at 25 °C for 5–7 days. At the time of inoculation, the colour of the medium was purple. The activity of isolates resulted in an acidification of the medium, turning it yellow. If the decarboxylase activity was positive, the agar surrounding the colonies turned purple.

#### 2.3.7. Amino Acid Decarboxylation Activity 

Petri dishes containing YPD agar supplemented with 1% (*w/v*) of the tested amino acid and 0.006% (*w/v*) bromocresol purple (Sigma-Aldrich) were prepared [15]. The amino acids analysed, L-ornithine (Panreac), L-lysine (Merck, Darmstadt, Germany), L-leucine (Merck), L-histidine (Panreac), L-phenylalanine (Panreac), L-arginine (Sigma-Aldrich), L-tryptophan (Merck) and S-tyrosine (Merck) were dissolved in water. Each prepared amino acid solution was mixed with the remaining fraction of medium, and pH was adjusted to 6.0. A single colony was spread onto the plates and was incubated at 25 °C for 7–14 days. The basis for the detection of the decarboxylase activity was the same as described for HCDC activity. 

### 2.4. Statistical Analysis

In order to analyse the distribution of the *S. cerevisiae* populations, the Shannon index ($H^{\prime }=-\sum_{i=1}^{S}\;p_{i}\ln\left(p_{i}\right)$) was calculated [5,25]. This index allowed us to relate the number of different strains (richness, S) to the number of isolates belonging to each strain (relative abundance, $p_{i}$), estimating the diversity in the yeast communities (α diversity). Differences between the obtained Shannon index values were established by analysis of variance (ANOVA), and, when the differences were statistically significant, the Tukey’s test was calculated.

In the same way, to describe the composition of the yeast populations, taking into account the variables vineyard (V1, V2 and V3) and vintage (first and second), the non-metric multidimensional scaling (NMDS) exploratory method was used. Using the raw abundance data, the Hellinger transformation was applied to reduce the effect of the many zeros in the data set. Subsequently, a matrix of object (strains) dissimilarities was calculated based on the Bray–Curtis distance. Then, the distances between the strains were ranked and mapped in two-dimensional ordination space by the NMDS algorithm [26,27]. This ordination method allowed us to visualise and compare the patterns of the strain distribution in each yeast community, distinguishing common strains shared by two or more populations and characteristic strains of a particular population (β diversity). Finally, a non-parametric PERMANOVA (permutational multivariate ANOVA) test was used to confirm if the differences observed in the distribution of the strains within the yeast communities were statistically significant. 

Cluster analysis was used to group the yeast strains according to the results of enzymatic activities. Ward’s method was applied on the squared Euclidean distances matrix and allowed us to compute the similarity among the strains. 

All the statistical analyses were carried out using the programmes IBM SPSS Statistics (v.26.0) and Statgraphics Centurion (v.19, Statgraphics Technologies, Inc. The Plains, VA, USA). Ecological diversity indexes and distances matrices were calculated utilizing PAST software [28].

## 3. Results and Discussion

### 3.1. Identification and Genetic Classification of Yeasts

The dynamic of yeast populations throughout alcoholic fermentation of Verdejo grapes from different vineyards and vintages was evaluated. *S. cerevisiae* isolated at different stages of alcoholic fermentation in the winery were subjected to an ecological study carried out at strain level. This approach required culture-dependent molecular techniques in order to establish microbial diversity considering genetic groups within species. Restriction analysis of mtDNA is considered one of the most convenient molecular tools for strain-level identification of *S. cerevisiae* [29,30]. Furthermore, this method has been widely used in the molecular characterisation and authentication of commercial strains, studies of population dynamics during spontaneous fermentations, analysis of yeast establishment in controlled fermentations and detection of wine spoilage yeasts [31]. Molecular identification and subsequent classification were carried out on a total of 484 isolates (Table 1). 

**Figure 1:**
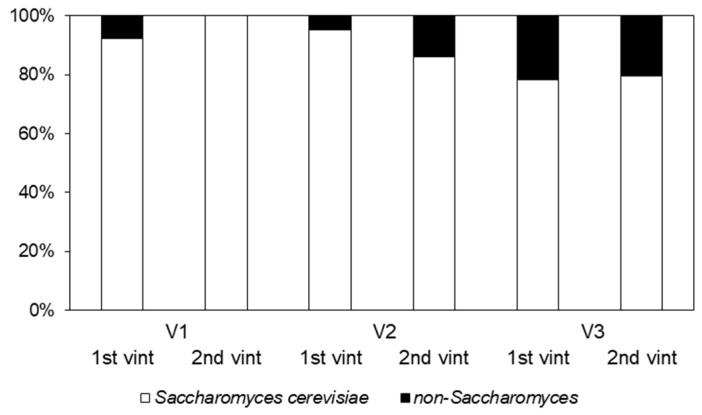
*S. cerevisiae* and non-*Saccharomyces* distribution across the vineyards (V1, V2, V3) and the first and second vintage (1st vint, 2nd vint).

The analysis of isolates at species level was performed, revealing that 89% (429 isolates) of them corresponded to *Saccharomyces* yeasts (Figure 1). The remaining isolates were identified as non-*Saccharomyces* yeasts, finding variable abundance between vineyards and vintages, with a higher presence of these species in vineyard 3 (21–22%). As expected, non-*Saccharomyces* yeasts were mainly found in the CM and RM stages, but a few isolates were also detected in the other stages of the fermentation process.

**Figure 2:**
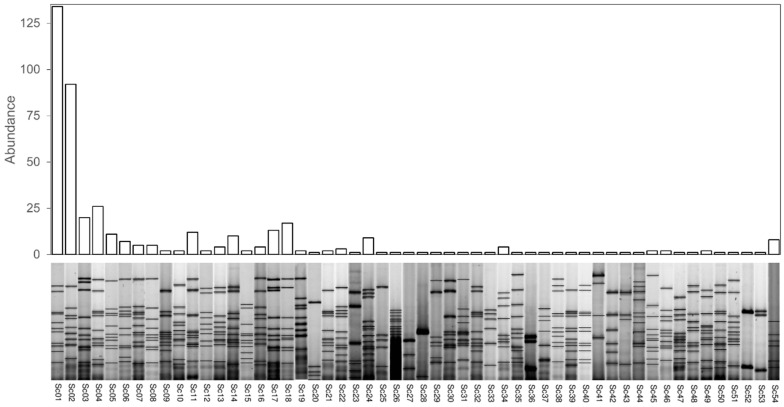
*S. cerevisiae* operational taxonomic units (OTUs) abundance determined by mtDNA–RFLP analyses and mtDNA patterns obtained with the *Hinf*I enzyme which identified each group.

The isolates corresponding to *S. cerevisiae* species were classified comparing their mtDNA restriction patterns, establishing 54 different genetic groups designated as operational taxonomic units (OTUs) (Figure 2). In concordance with these results, previous studies, which focused on identification of *S. cerevisiae* strains by mtDNA-RFLP technique in wineries throughout spontaneous fermentation, revealed a high diversity of *S. cerevisiae* populations [5,22]. The distribution of the isolates in each OTU was not homogeneous, with more than half of the isolates belonging to OTUs Sc01 and Sc02 (134 and 92 isolates, respectively). The remaining isolates were distributed as follows: 6 OTUs were formed by a number of isolates between 11 and 26, 10 of them included between 3 and 10, 9 OTUs contained 2 isolates and 27 OTUs were represented by one isolate (Figure 2).

Relative abundance of a strain above 10% has been considered by other authors as a dominant strain [5]. Taking into consideration the distribution and relative abundance of the described OTUs, Sc01 and Sc02 were considered as dominants during spontaneous fermentation, as their relative abundance widely exceeded this percentage, independently of vineyard or vintage.

### 3.2. Analysis of Diversity of S. cerevisiae Population

**Figure 3:**
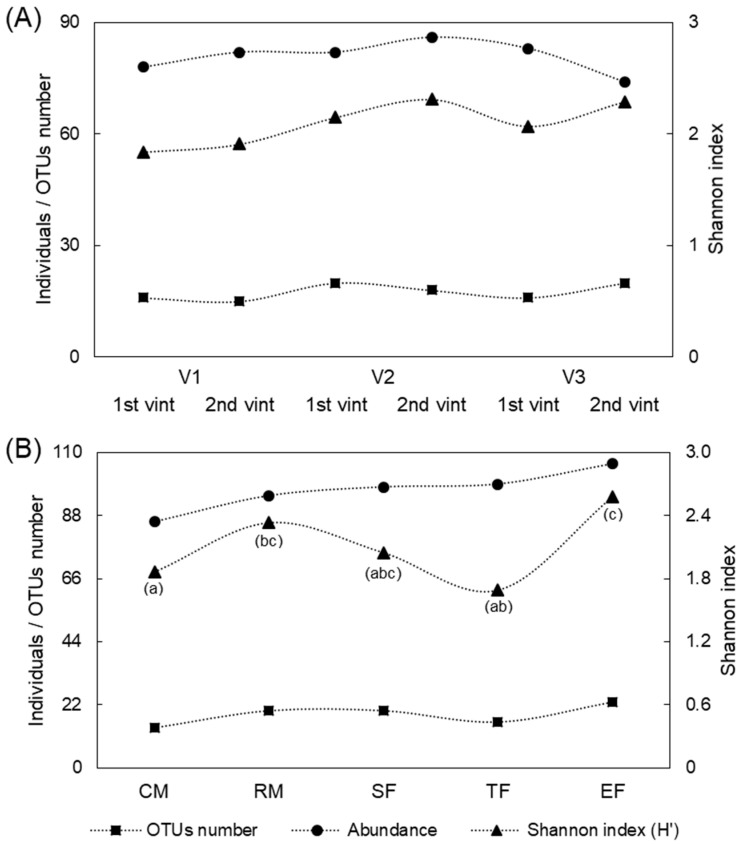
Yeast community α diversity based on OTUs number (richness), number of isolates belonging to each OTU (abundance) and Shannon index. (**A**) Vineyard (V1, V2, V3) and vintage (1st vint, 2nd vint) diversity. (**B**) Fermentation stages diversity (freshly crushed grape must, CM; racked must, RM; start of fermentation, SF; tumultuous fermentation, TF; end of fermentation, EF). Different letters indicate significant differences among different stages of the winemaking process.

Geographical and temporal diversity of *S. cerevisiae* populations were determined according to total populations isolated from the vineyards (V1, V2 and V3) in both vintages. The Shannon index was calculated to estimate the diversity of *S. cerevisiae* populations found (α diversity) in each winemaking process [32,33]. The values obtained were compared by one-way ANOVA in order to determine differences between *S. cerevisiae* populations. Likewise, the sequential yeast populations found throughout the stages of alcoholic fermentation (CM, RM, SF, TF and EF) were evaluated (Figure 3).

The comparison of diversity indexes showed no significant differences in *S. cerevisiae* distribution patterns between vineyards or vintages (vintage *p*-value = 0.384; vineyard *p*-value = 0.773) (Figure 3A). These results illustrate the similar distribution of isolates in winemaking processes from different geographical localisations or vintages, defining a common structure where *S. cerevisiae* populations are mainly represented by two OTUs (Sc01 and Sc02), and most of the remaining OTUs comprise one to three isolates. In agreement with the diversity index values obtained in this research, the study of the diversity of *S. cerevisiae* in five organic wineries from the northwest of Spain revealed similar results. A wide distribution of some strains was found in several wineries but also the presence of winery-specific strains contributing to wine differentiation was found [5]. Although there is evidence of geographical influence in *S. cerevisiae* population, differences in microbial diversity at small scales is not clearly defined [34]. Similar to our study, Knight et al. did not find significant differences in species richness in fungal communities among vineyards in soil samples [34].

However, the analysis of population patterns revealed significant differences at different stages of the fermentation process (*p*-value = 0.001). Tukey’s test confirmed differences between *S. cerevisiae* populations presented in the CM stage and RM and EF stages and between the TF and EF stages. Both the CM stage and TF stage showed the lowest diversity values (1.86 and 1.69, respectively), while the RM and EF stages presented the highest diversity values (2.33 and 2.58, respectively) (Figure 3B).

These results confirmed the expected development of the *S. cerevisiae* population during alcoholic fermentation [35]. On the one hand, the CM stage presented lower abundance and richness of *S. cerevisiae* strains as oxidative yeasts were demonstrated to be predominant at this stage [31]. On the other hand, although the TF stage is characterised by a high abundance of *S. cerevisiae*, a lower richness of strains was found. In contrast, a high diversity was found before the beginning (RM stage) and at the end of fermentation (EF stage). Richness of *S. cerevisiae* found at the different stages of the winemaking process, mainly the differences found between the CM and RM stages, can be explained in accordance with previous studies which confirmed the influence of the air and winery environment in spontaneous fermentations. Thus, resident strains of *S. cerevisiae* at the winery may be well adapted and easily implanted compared to those from the grape [5,36,37].

### 3.3. Spatial and Temporal Concurrence of S. cerevisiae Strains 

**Figure 4:**
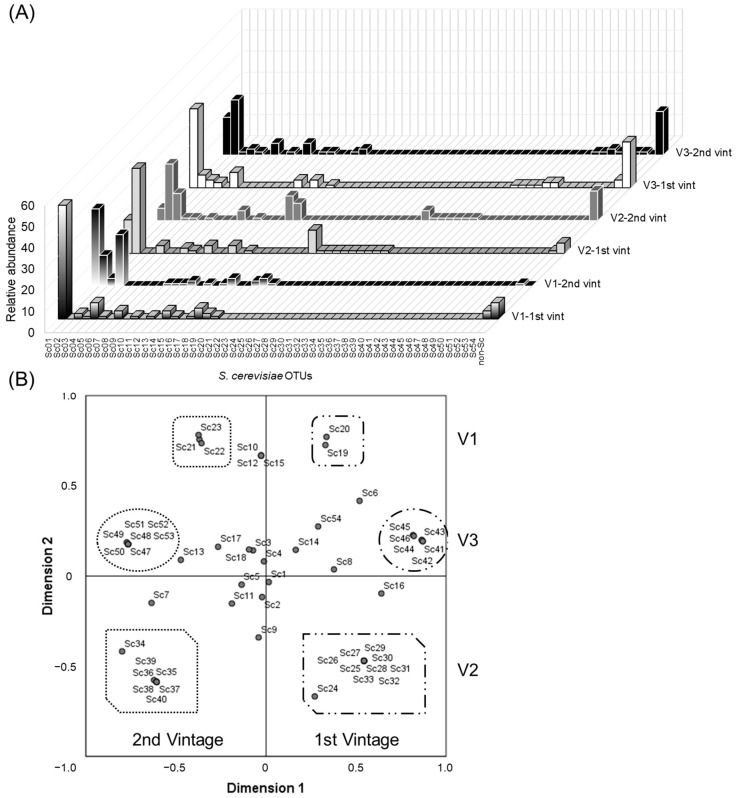
β diversity (yeast community dissimilarity). (**A**) *S. cerevisiae* populations established across the vineyards (V1, V2, V3) and the vintages (1st vint, 2nd vint). (**B**) Non-metric multidimensional scaling (NMDS) using Bray–Curtis distance. The S-stress value was 0.00772. The groups of strains representative of each vineyard population (V1, V2, V3) are delimitated by circles or rectangles forms, and the vintage populations (first and second) are distinguished by the different border in the forms (dots or dots and lines, respectively). Significant differences were found among samples (*p*-value = 0.0001).

Distribution and fluctuation of *S. cerevisiae* strains across vineyards and vintages are represented in Figure 4A. Aiming for a better overview, the non-metric multidimensional scaling (NMDS) technique was applied. Other authors pointed at this exploratory method of organisation as an appropriate tool for microbial ecology studies based on molecular fingerprinting [26,27]. NMDS analysis allowed a graphic display of the existing relationship among the strains which defined the six populations of yeasts analysed according to the variables vintage and vineyard (Figure 4B). Thus, the diversity of unique OTUs (β diversity), which reflects the composition of the communities, was established. 

As shown in Figure 4B, the NMDS dimensions determined the divergence among OTUs according to the surveyed vintages and vineyards. The subset of the first vintage was located at an NMDS dimension 1 > 0, while the subset of the second vintage was located at a dimension 1 < 0. Likewise, dimension 2 defined the divergence of subsets vineyard V1 (dimension 2 > 0.5), V2 (dimension 2 < 0) and V3 (0 < dimension 2 < 0.5). The stress value obtained (0.00772) was indicative that the NMDS model correctly represented the original populations. Thus, six groups of OTUs were defined as unique and representative of each of the populations analysed. It was noticed that more than 77% of the representative OTUs of each vineyard comprised only one isolate. The statistical significance of the differences observed in the NDMS analysis was confirmed by PERMANOVA analysis (*p*-value = 0.0001). 

Previous studies pointed at climate conditions and soil environments as influencing factors in microbial communities and, thus, in wine quality and regional characteristics [4]. Focusing attention on meteorological conditions in both vintages, differences between them were found (Figure S1). On the one hand, the first vintage presented a normal distribution of precipitation during the year, with a behaviour concordant with the usual rainfall patterns. However, the second vintage recorded less precipitation and an uneven distribution of the volume of precipitation. On the other hand, temperature values throughout the second vintage were more irregular. Regarding the vineyards in this study, there were differences in soil type, orientation and year of plantation (Table S1). In this sense, the effect on the pattern of yeasts associated with grapes from vineyards has also been addressed previously by other authors [33].

Taken together, these results highlight the effect of vineyard and vintage on yeast communities, as well as the presence of singular strains in each vineyard and vintage. 

### 3.4. Evaluation of the Contribution of Yeast Populations to Wine Quality 

Enzymatic activities related to wine quality were determined in the 54 OTUs of *S. cerevisiae* found in this study. On the one hand, there were those related to organoleptic profile (β-lyase, β-glucosidase, sulfite reductase and HCDC activities) and β-glucanase and protease activities (also involved in fining). On the other hand, there were those related to the production of BA (amino acid decarboxylase activity).

**Figure 5:**
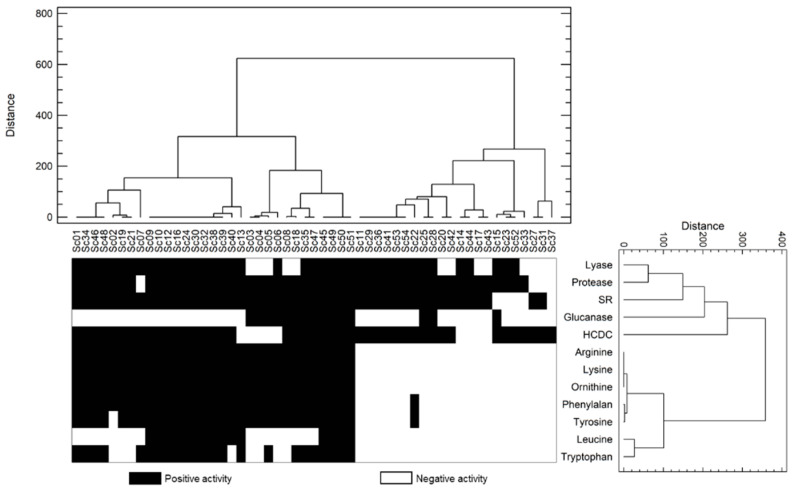
Enzymatic activities found in the representative isolates of each strain were represented by cluster analysis based on Ward´s method and squared Euclidean distances matrix. The isolates are grouped based on the positive or negative enzymatic activities shown. A dendrogram allows us to relate isolates with similar enzymatic activity profiles. The enzymatic activities determined in each isolate are represented by a heat map.

Differences among isolates were found in all the enzymatic activities analysed except for β-glucosidase and histidine decarboxylase activities. None of the 54 tested isolates showed growth on the β-glucosidase selective medium, showing a lack of this activity. Accordingly, previous studies have stated that *S. cerevisiae* strains lack β-glucosidase activity, associated mainly with non-*Saccharomyces* yeasts [15,24,38]. Regarding the histidine decarboxylase assay, the results were not conclusive, as no changes in the colour of the growth medium were observed in any of the tests carried out. As oenological factors have a crucial influence on BA production [17], further studies on real winemaking conditions are under consideration. Therefore, both activities were not included in the subsequent cluster analysis. The remaining activities were positive in more than 50% of the isolates, except β-glucanase activity (28.3%) and leucine (28.3%) and tryptophan (43.4%) decarboxylase activities, which showed lower percentages of isolates with positive enzymatic activity (Figure 5). Despite the differences in the enzymatic activity of the strains, no relation was established relating to isolate origin (vineyard, vintage or fermentation stage).

Potential BA-producing strains were also determined. Focusing on the group of isolates without or with very low potential to produce BA (41.5% of isolates), a high percentage of positive isolates was detected with β-lyase (63.6%), protease (86.4%), sulfite reductase (77.3%) and HCDC (81.8%) activities. A moderate percentage (13.6%) showed β-glucanase activity. Despite the rest of the isolates presenting the potential ability to produce BA, oenologically relevant activities such as β-lyase, protease and β-glucanase were detected in 83.9%, 96.8% and 38.7% of the isolates, respectively. Additionally, non-desired activities, sulfite reductase and HCDC, were also detected in a high percentage of the isolates (100% and 83.9%, respectively).

Taken together, the strains analysed in this study showed interesting positive activities, contributing collectively to the final characteristics of wine to different extents. In contrast to our data, Tristezza et al. [15] found a poor positive enzymatic potential in *S. cerevisiae* strains, as β-glucosidase and protease were not detected, while a few isolates presented β-glucanase activity. In the same way, our results demonstrated positive β-lyase activity in 75.5% of the total isolates studied in contrast to previous data where *S. cerevisiae* strains were found to be low varietal thiol producers, as β-lyase activity is mainly associated with non-*Saccharomyces* yeasts [12]. The dominant OTUs Sc01 and Sc02 showed interesting enzymatic activities, such as β-lyase and protease, although they also presented a potential capacity for amino acid decarboxylation. Of all the non-dominant isolates, only OTU Sc15 showed positive activity in all the enzymes with favourable impact on the organoleptic profile of the wine and negative activity on the enzymes with unfavourable impact, except for HCDC activity. In a similar way, OTUs Sc23 and Sc52 displayed the same activity profile as Sc15, except for β-glucanase activity, which was negative in both. Further studies are under consideration to test the effect of these isolates on the final composition of wine.

As spontaneous fermentation is a complex microbial process in which several species/strains of non-*Saccharomyces* and *Saccharomyces* yeasts take part, understanding the interactions between yeast populations is a key step to understanding the contribution of each strain to the grape–wine ecosystem. Cell-to-cell communication modulates metabolic processes such as the production of enzymes and metabolites, among other things, impacting the final composition and organoleptic properties of wine [39,40,41]. Further studies need to be performed in this way to understand the role of well-adapted strains common to most populations and unique strains within a population in achieving their maximum expression in the winemaking process.

## 4. Conclusions

In the present study, evaluation at strain level within *S. cerevisiae* populations was carried out, evidencing that spontaneous fermentations are complex ecosystems. Taken together, the results demonstrate the high diversity of the *S. cerevisiae* populations involved in the spontaneous winemaking process. The analysis of distribution and fluctuation of the yeast communities in vineyards and vintages suggest a geographical and temporal influence on *S. cerevisiae* populations for each vineyard and vintage. Furthermore, the variability in the enzymatic activities of the strains highlights the significance of organic viticulture being carried out in conjunction with spontaneous fermentation in order to preserve the microbial diversity and distinctiveness of Verdejo wine. Overall, the results obtained in this study are the first step to emphasise the importance of maintaining microbiota variability in the quality and uniqueness of organic Verdejo wine and would be of potential interest in future studies hoping to define the role of specific species in the organoleptic complexity of wine. Further research describing interactions between species/strains of the same community involved in the typicity in wines will help us to understand and preserve biodiversity as a biotechnological tool in the winemaking process.

## Figures and Tables

**Table 1:** Number of yeasts isolates analysed in this work, depending on the vineyard, vintage and fermentation stage.

Fermentation Stages	Vineyard 1		Vineyard 2		Vineyard 3		Total
	1st vint	2nd vint	1st vint	2nd vint	1st vint	2nd vint	
CM	13	14	16	15	16	12	86
RM	15	17	16	17	16	14	95
SF	16	17	17	16	17	15	98
TF	17	17	16	17	17	15	99
EF	17	17	17	21	17	17	106
Total	78	82	82	86	83	73	484

Fermentation stages: freshly crushed grape must, CM; racked must, RM; start of fermentation, SF; tumultuous fermentation, TF; end of fermentation, EF. Vintages: 1st vint and 2nd vint.

## Data Availability

Data are contained within the article or Supplementary Materials.

## Supplementary Materials

The following supporting information can be downloaded at: https://www.mdpi.com/article/10.3390/foods11213448/s1, Figure S1: Maximum, minimum and average temperatures and monthly precipitation registered in the period of time that defined the first and second vintages. The accumulated precipitation during this time was 393.3 mm and 217.0 mm, respectively. InfoRiego database: www.inforiego.org (accessed on 9 October 2022) and ITACyL database: http://www.itacyl.es/agro-y-geo-tecnologia/agrometeorologia-y-suelos/datos-meteorologicos (accessed on 9 October 2022), for the Rueda weather station (VA103), Valladolid; Table S1: Description of the vineyards of this study.
