# Supplementary material for: Screening and Enzymatic Evaluation of Saccharomyces cerevisiae Populations from Spontaneous Fermentation of Organic Verdejo Wines
Source: Foods. 2022 Oct 30;11(21):3448. doi: 10.3390/foods11213448 (PMC9656934; doi:10.3390/foods11213448)
Supplement: Supplementary file 1 [file foods-11-03448-s001.zip › foods-1990640-supplementary.pdf]

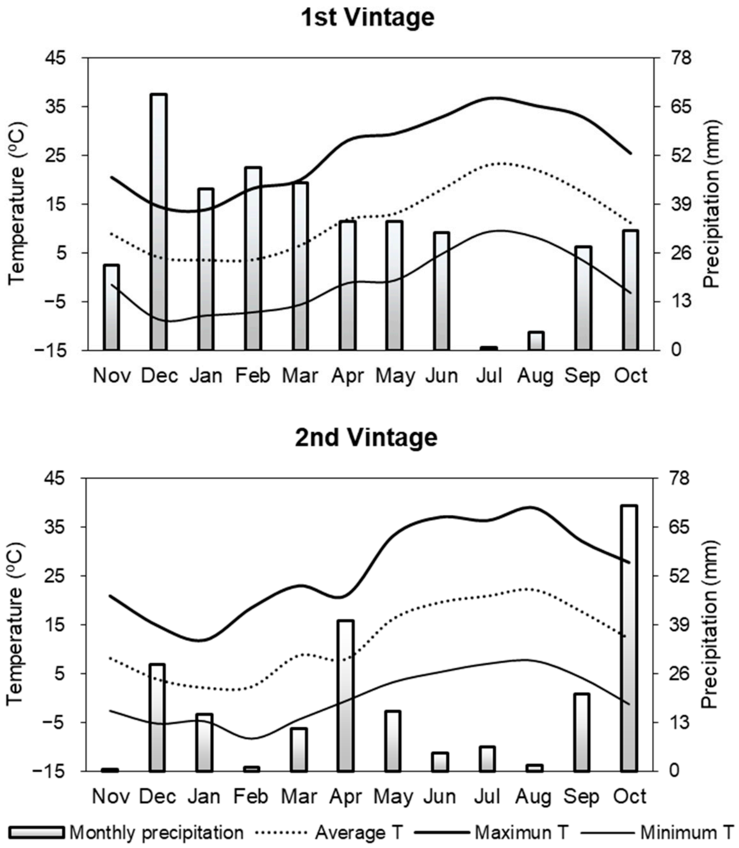

Figure S1. Maximum, minimum and average temperatures and monthly precipitation registered in the period of time that defined the first and second vintages. The accumulated precipitation during this time was 393.3 mm and 217.0 mm, respectively. InfoRiego database: www.inforiego.org and ITACyL database: http://www.itacyl.es/agro-y-geo-tecnologia/agrometeorologia-y-suelos/datos-meteorologicos, for the Rueda weather station (VA103), Valladolid.

Table S1. Description of the vineyards of this study.

Description item	Vineyard 1	Vineyard 2	Vineyard 3
Plantation year	1999	1999	2005-2006
Variety	Verdejo	Verdejo	Verdejo
Plantation density (grapevines/ha)	1111	1111	3333
Training system	Trellis	Trellis	Trellis
Soil type	Gravel-clay-sandy	Gravel-clay	Gravel-clay-limestone
Orientation	S	S	SW
Orography	Plateau	Plateau	Plateau
